# Comparative study of the efficacy of herbal antioxdants oxitard and aloe vera in the treatment of oral submucous fibrosis

**DOI:** 10.4317/jced.51424

**Published:** 2014-07-01

**Authors:** Santosh Patil, Vishal Halgatti, Sneha Maheshwari, B S. Santosh

**Affiliations:** 1 Reader, Dept of Oral Medicine and Radiology, Chattisgarh Dental College Research Institute, Rajnandgaon, Chattisgarh. India; 2 Assistant Professor, Dept of Dentistry, Belgaum Institute of Medical Sciences, Belgaum,Karnataka. India; 3 Dental Practitioner, Jodhpur, Rajasthan, India; 4 Reader, Dept of Oral and Maxillofacial Surgery, Chattisgarh Dental College Research Institute, Rajnandgaon, Chattisgarh. India

## Abstract

Objectives: Oral submucous fibrosis (OSMF) is a potentially malignant disorder predominantly seen in the Indian subcontinent due to areca nut, tobacco and their products. The aim of the present study was to compare the efficacy of oxitard and aloe vera in the management of OSMF. Material and Methods: 120 subjects with OSMF were included in the study. The patients were clinico-pathologically diagnosed and divided equally in 2 groups, Group A (oxitard group) and Group B (aloe vera group). Group A was administered 2 oxitard capsules twice daily and Group B was given 5 mg aloe vera gel to be applied topically thrice daily for 3 months. Different clinical parameters were evaluated at regular intervals. Data was analyzed using the Student’s paired t test and Chi-square test. P-value <0.001 was considered to be statistically significant. Results: Clinical improvements in mouth opening and tongue protrusion was significant in the oxitard group (p=0.0005). Subjective symptoms of pain associated with the lesion (p=0.0003), difficulty in swallowing (p=0.0000) and speech (p=0.0001) also significantly improved in the Group A. The improvement in burning sensation was not statistically significant between the 2 groups (p=0.002). There was a mild to moderate decrease in the size of the lesion. Conclusions: Though there is no definitive treatment for the condition however, overall assessment of symptoms like mouth opening, tongue protrusion, difficulty in swallowing and speech and pain associated with the lesion showed that oxitard capsules can bring about significant clinical improvements than aloe vera gel in the treatment of OSMF.

** Key words:**Oral submucous fibrosis, oxitard capsules, aloe vera, burning sensation, mouth opening.

## Introduction

Oral submucous fibrosis [OSMF] is a high risk premalignant condition of oral cavity, pharynx and upper diges-tive tract, characterized by progressive inability to open the mouth and by inflammation and progressive fibrosis of the submucosal tissues (1). Susrutha described a condition similar to OSMF as “vidari”, under the umbrella of mouth and throat diseases in ancient medicine (2). Pindborg and his associates (3) defined the condition as “an insidious chronic disease affecting any part of the oral cavity and sometimes pharynx. Although occasionally preceded by and/or associated with vesicle formation, it is always associated with juxtaepithelial inflammatory reaction followed by fibroelastic changes in the lamina propria, with epithelial atrophy leading to stiffness of the oral mucosa causing trismus and difficulty in eating.” The pathogenesis of the disease is not well known, but the etiology is believed to be multifactorial. The condition is particularly associated with chewing irritants such as areca nut, which is the main component of betel quid, tobacco and their numerous products. The habit of betel quid chewing is practiced predominately in the Indian subcontinent from a long time (1). These trigger the synthesis of collagen, tough fibrinous protein which causes stiffening of the soft mucous membrane and muscles of the oral cavity. The tongue usually escapes as it is highly vascular. The mouth opening reduces and in extreme cases, only a button size opening is left (4). There is scarring with atrophy of the mucous membrane and pain during swallowing. The atrophic mucous may ulcerate often, and subsequently may lead to malignancy (5).

Treatment modalities both medical and surgical, for relieving the symptoms have been advocated, but have not been successful so far. Therefore, the search for an effective treatment modality still continues. Many natural plants extracts, synthetic drugs, etc have been introduced and tried for the management of OSMF. One such plant is aloe vera which promotes wound healing, and also has anti-inflammatory, immunomodulatory, and antioxidant properties (5). Oxitard is another herbal antioxidant formulation containing the extracts of *Mangifera indica, Withania somnifera, Daucus carota, Glycyrrhiza glabra, Vitis vinifera*, powders of *Emblica officinalis* and *Yashada bhasma*; and oils of *Triticum sativum* (6). The aim of the present study was to compare and evaluate the efficacy of herbal antioxidants oxitard and aloe vera in the management of OSMF.

## Material and Methods

The current prospective, randomized and single blind study included 120 OSMF patients reporting to the Department of Oral Medicine and Radiology, Jodhpur Dental College General Hospital. The patients were diagnosed clinically and histopathologically for OSMF. Patients of either sex with OSMF were included in the study. Ethical clearance was obtained from the Institutional Ethical Committee. A written informed consent was obtained from the patients prior to the inclusion in the study. Patients who were not taking any medications for OSMF previously were included in the study. Those with any evidence of hypersensitivity to aloe vera, severe psychiatric, cardiac, gastrointestinal or metabolic disorders, pregnancy and lactation were excluded from the study. Detailed family and medical history and history of any associated habits and the course of the disease was recorded. A thorough clinical examination was carried out by oral medicine specialist and relevant findings were recorded. The subjects were also advised and encouraged to quit the associated habit during the study period. The subjects were randomly divided in 2 equal groups, Group A [oxitard group] and Group B [aloe vera group]. Group A was administered 2 oxitard capsules twice daily and Group B was given 5 mg aloe vera gel [Sheetal Lab, Surat] to be applied topically thrice daily for 3 months and were further followed up for a period of 2 months. Patients were advised not to eat or drink for 15 minutes after the application of the aloe vera gel. Mouth opening was measured by measuring the distance between the centre of incisal edges of maxillary central incisors and mandibular central incisor at maximum opened mouth. In edentulous patients, the inter ridge [alveolar] distance along the midline was measured (7). Three measurements were recorded consecutively and the average value was calculated and recorded. Tongue protrusion was measured as distance between lower central incisor and tip of the tongue on protrusion (7). Evaluation for presence, absence or reduction of other clinical parameters such as burning sensation, pain associated with the lesion, difficulty in swallowing and speech and variation in the size of the lesion, which was assessed based on the blanching of the oral mucosa was done at regular intervals of 1 month, 2 month and 3 months. The data was entered using computer software SPSS 12.0 [SPSS Inc., Chicago, USA] and analyzed using the Student’s paired t test and Chi-square test. P-value<0.001 was considered to be statistically significant.

## Results

There were 64 males and 56 females with a mean age of 31.6±12.7 years. 56% of the patients had habit of betel nut chewing, while 26% of the patients had tobacco chewing habit. 40% of the patients consumed spicy foods which were among the main causative factors for OSMF in the study population. Clinical improvements in mouth opening and tongue protrusion was significant in the Group A [p=0.0005] ( Table 1, Table 2). The effect of administration of oxitard capsules in the Group A showed significant improvement in the subjective symptoms of pain associated with the lesion [p=0.0003], difficulty in swallowing [p=0.0000] and speech [p=0.0001] when compared to the Group B who were given aloe vera (Table  Table 3- Table 5). However, there was no significant improvement in burning sensation [p=0.002] among the 2 groups. There was a mild to moderate decrease in the size of the lesion. Nineteen patients of Group A and ten patients from Group B showed a severe degree of change in the size of the lesion, i.e., a decrease of >2.5 cm. Twenty one patients from Group A and twenty one patients from Group B showed a moderate [1.5-2.5 cm] change in the size of the lesion at the end of the study. Fifteen patients from Group A and nineteen patients from Group B showed a mild [0-1.5 cm] change in the size of the lesion. The improvement was not significant [p>0.001] among the 2 groups. However, five patients from Group A and ten patients from Group B showed no change in the lesion size over the study period. Eight patients experienced mild abdominal discomfort due to oxitard. There were no reported side effects. There were no dropouts from the study due to any reason during the follow-up. For oxitard group, during follow-up changes were similar to the results at the treatment except for 5 cases that showed pain and burning sensation of oral cavity for a period of 10 days. However, 16 patients from the aloe vera group reported pain, burning sensation and mild increase in the size of the lesion during the follow-up period.

**Table 1:**
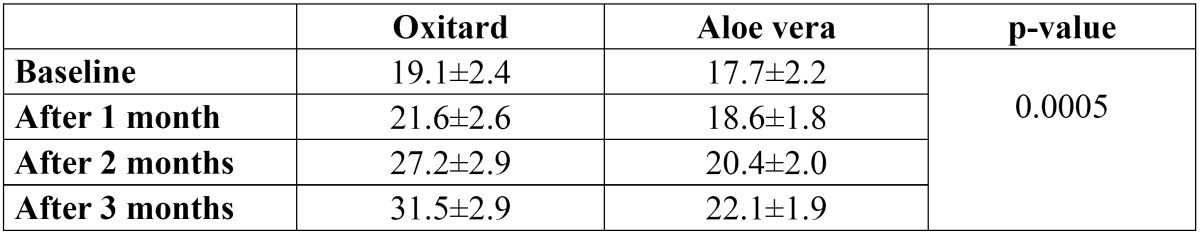
Effect of oxitard and aloe vera in improving mouth opening (mean values in mm).

**Table 2:**
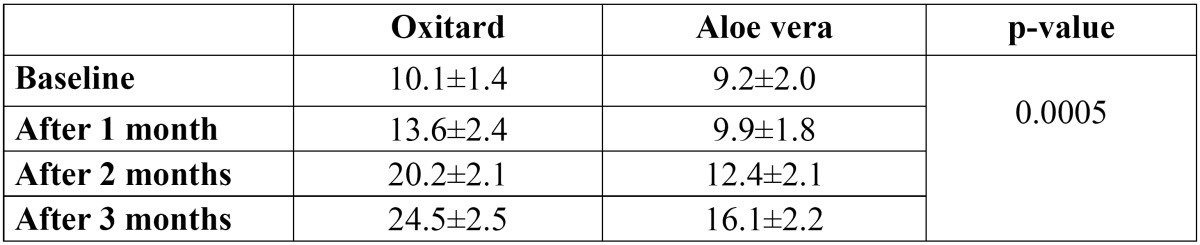
Effect of oxitard and aloe vera in improving tongue protrusion (mean values in mm)

**Table 3:**
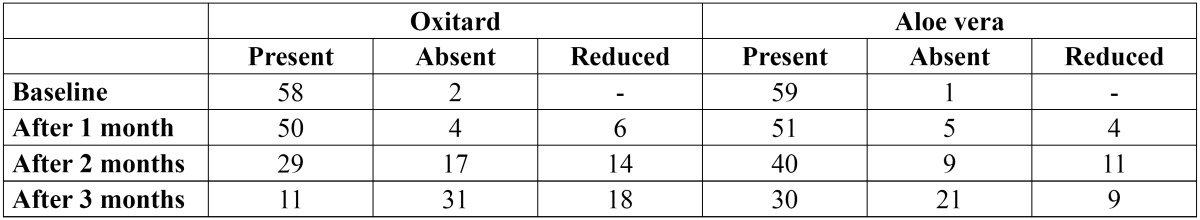
Effect of oxitard and aloe vera on pain associated with the lesion.

**Table 4:**
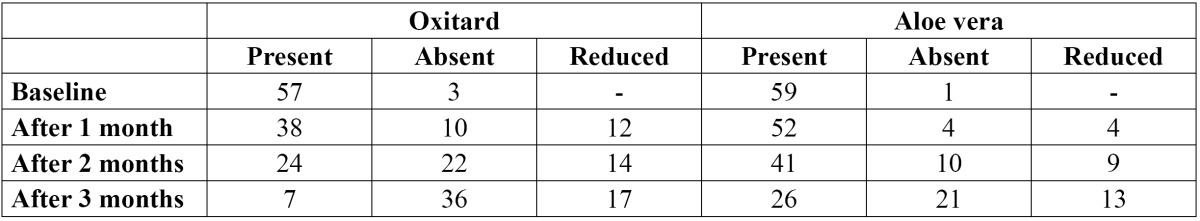
Effect of oxitard and aloe vera on difficulty in swallowing.

**Table 5:**
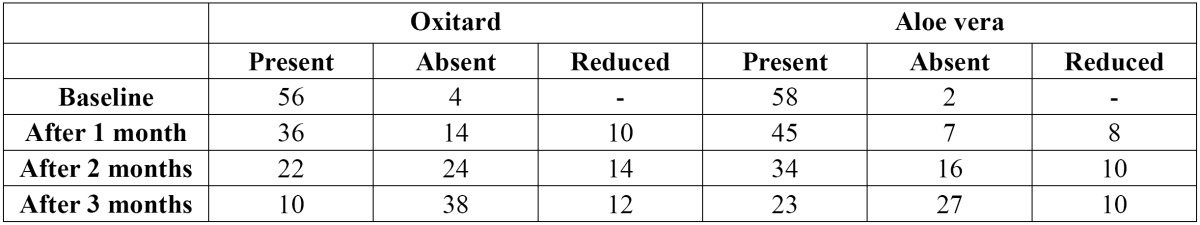
Effect of oxitard and aloe vera on difficulty in speech.

## Discussion

OSMF is a chronic disease of the oral cavity and oropharynx which is predominantly seen in the Indian subcontinent and Southeast Asian countries, commonly seen in the patients chewing arecanut and is now globally considered an Indian disease. The overall prevalence rate in India is estimated to be about 0.2% to 0.5% and preva-lence by gender varying from 0.2-2.3% in males and 1.2-4.57% in females (8,9). It is considered to have a high degree of malignant potential, which ranges between 2.3% and 7.6% (10). The condition is characterized by burning sensation of the oral mucosa, ulceration and pain, blanching of oral mucosa, reduced movement and depapillation of tongue, depigmentation of oral mucosa, and progressive reduction of mouth opening (11,12). Advanced stages of the condition may be characterized by nasal twang due to fibrosis of nasopharynx and hearing impairment due to stenosis of eustachian tube. The pathophysiology is complex, and various factors such as, ingestion of chilies, genetic susceptibility, nutritional deficiencies, altered salivary constituents, autoimmunity and collagen disorders may be involved in the pathogenesis (8). Areca nut and its products are the most common etiological factor. Majority of OSMF patients present with irreversible moderate-to-severe condition. The changes of OSMF are limited to oral tissues and similar to those of scleroderma. It may be associated with oral leukoplakia and other potentially malignant disorders or with malignancy such as squamous cell carcinoma (11). The precancerous nature of OSMF has been proved by, higher occurrence of OSMF in oral squamous cell carcinoma patients, histological diagnosis of cancer without any clinical suspicion in OSMF, high frequency of epithelial dysplasia and higher prevalence of leukoplakia among OSMF. The debate over the initiation of malignancy in OSMF due to epithelium or due to connective tissue is still unanswered (9,13). However, it has been suggested that the pathology develops within the epithelium due to intraoral trauma and various factors such as, irritation from jagged teeth, sharp overhanging restoration, ill-fitting dentures, jacket crowns, prolong use of tobacco and poor oral hygiene (14).

Many therapeutic and surgical treatment modalities for relieving the symptoms have been advocated, but no definitive and widely accepted treatment is currently available. The first step of preventive measure should be in advising the patient to discontinue the habit, which can be encouraged through education, counseling and advocacy. Medical treatment includes steroids, placental extracts, IFN γ, lycopene, pentoxifylline, surgical excision, laser removal, etc. These have proved to be symptomatic and are predominantly aimed at improving mouth movements. But each treatment has its own limitations. According to Caniif *et al.* (10) the medical management of OSMF is both empirical and unsatisfactory. According to Rehana *et al.* (15) multiple minerals and micronutrients showed significant improvement in mouth opening of 41% of the patients. Whereas, Borle *et al.* (16) showed improvement in symptoms of OSMF but insignificant improvement in mouth opening with vitamin A. Lycopene has also showed significant improvement in mouth opening in the study by Karemore *et al.* (17). Singh *et al.* have shown significant improvement in mouth opening, hyperkeratosis, pain in mouth and size of the lesion with oxitard capsules (6). Sudarshan *et al.* (18) have shown significant improvement in the mouth opening with aloe vera. No studies citing the comparative effectiveness specifically of oxitard and aloe vera in the treatment of OSMF have been done till date. Hence, the present study compared the efficacy of the 2 herbal antioxidants oxitard and aloe vera in the improvement of various clinical parameters such as, mouth opening, difficulty in swallowing, speech, pain associated with the lesion, tongue protrusion and burning sensation.

The formulation of the oxitard capsules contains the extracts of Mangifera indica, Withania somnifera, Daucus carota, Glycyrrhiza glabra, Vitis vinifera, powders of Emblica officinalis and Yashada bhasma; and oils of Triticum sativum. Mangifera indica is shown to have antibiotic, anti-asthamatic, antiseptic, antiviral, hypotensive, anti-emetic properties. Withania somnifera provides overall health and wellness with its anti-stress, anti-anxiety, anti-inflammatory, anti-convulsive and anti-arthritic properties. Daucus carota acts as a good antiseptic as it is a rich source of vitamin A. Glycyrrhiza glabra normalizes the hoarseness in voice and has immunomodulatory and anti-inflammatory properties. Vitis vinifera have anti-inflammatory, astringent and an effect to curb the burning sensation. Emblica officinalis is a rich source of vitamin C and is a potent antibiotic. Yashada bhasma contains zinc which plays a significant role in protein synthesis, cell division and wound healing. Triticum sativum is a rich source of minerals and has an antioxidant property (6).

Aloe vera is a mannoprotein containing many amino acids known as ‘wound healing hormones’. The polysaccharides contained in the gel of the leaves, promote wound healing, and have anti-inflammatory, immunomodulatory, antioxidant properties and gastro protective properties. Further, sterols in the Aloe vera have strong ability to inhibit inflammation similar to the action of cortisone without any side effects. It can be found easily and is of low cost in India (18).

Till date, no treatment protocol has been formulated to restore the mouth opening to normal; however an improvement of a few millimeters has been observed. The present study showed improvement in both the groups at the end of the study. The results were much better in the oxitard group when compared to the patients who were advised to apply aloe vera gel locally. There were significant improvements in mouth opening [p=0.0005], tongue protrusion [p-0.0005], pain associated with the lesion [p=0.0003], difficulty in swallowing [p=0.0000] and speech [p=0.0001] in the oxitard group when compared to the aloe vera group. However, the improvements in burning sensation were not statistically significant [p=0.002].

The present study showed that mouth opening improved 60.5% in Group A, whereas Group B showed 25% improvement. This was not in line with the findings of Sudarshan *et al.* (18) who showed an improvement of 20% in the patients who were given aloe vera, whereas those who were given antioxidants showed an improvement of only 9%, which was much lower than the findings of the present study. Singh *et al.* (6) showed a significant [p<0.05] improvement in mouth opening with oxitard capsules. Sudarshan *et al.* (18) showed an improvement of 80% in burning sensation in the aloe vera group whereas 65.7% patients showed in the antioxidant group showed improvement in burning sensation. This difference was statistically significant [p=0.008], in contrast to the findings of the present study where the difference in the improvement in both the groups was not statistically significant [p=0.002]. Biweekly submucosal injection for a period of 10 weeks with dexamethasone [4 mg], hyaluronidase [1500 IU], and placental extracts [2 ml] also showed similar results in the 82%, 82%, 51% reduction in burning sensation, respectively (19). Improvement in tongue protrusion was significantly [p=0.0005] higher in the Group A than Group B. Sudarshan *et al.* (18) showed an improvement of 8.8% in the aloe vera group while the antioxidant group showed 4.25% improvement.

The present study also showed a significant improvement in the pain associated with the lesion [p=0.0003], difficulty in swallowing [p=0.0000] and speech [p=0.0001]. Thirty one patients in the Group A and 21 patients in Group B showed complete absence of pain at the end of the treatment. While in the study conducted by Singh *et al.* (6) none of the patients who were administered oxitard capsules showed severe degree of pain in OSMF patients. Only 7 patients in Group A had difficulty in swallowing at the end of the study, whereas 26 patients still complained of difficulty while swallowing in Group B. Ten patients in Group A complained of difficulty in speech, while 26 patients in Group B had persistent difficulty in speech at the end of 3 months. There was a mild to moderate decrease in the size of the lesion. Ninteen patients of Group A and 10 patients from Group B showed a severe degree of change in the size of the lesion. 21 patients from both the groups showed a moderate [1.5-2.5 cm] change in the size of the lesion at the end of the study. Fifteen patients from Group A and 19 patients from Group B showed a mild [0-1.5 cm] change in the size of the lesion. Five patients from Group A and 10 patients from Group B showed no change in the lesion size at the end of 3 months. The study conducted by Singh *et al.* (6) showed that 29 patients showed no improvement at the end of the study, while only 3 patients showed severe [>2 cm] improvement in the size of the lesion.

Oxitard has shown significant improvement in mouth opening, tongue protrusion, pain with the lesion, difficulty in swallowing and speech when compared to aloe vera. Aloe vera has shown anti-inflammatory properties over a wide range of experiments. The sterols in Aloe have strong anti-inflammatory properties, similar to cortisone, without any of the side effects. The fact that aloe works similar to aspirin in blocking prostaglandin effects, still remains to be proven. Aloe vera can be applied topically, is easily available, safe to use, cost effective, non-invasive and effective treatment modality for OSMF. Both the medicaments can be considered to be an effectual protocol in the management of OSMF. However, the results with oxitard capsules were significantly better than the aloe vera group. Quitting of the habit alone as an intervention may have a significantly greater effect, on the symptoms of OSMF. Hence, intervention studies and public health campaigns at the community level may be the best way of controlling OSMF.
